# The Triad Mother-Breast Milk-Infant as Predictor of Future Health: A Narrative Review

**DOI:** 10.3390/nu13020486

**Published:** 2021-02-02

**Authors:** Elvira Verduci, Maria Lorella Giannì, Giulia Vizzari, Sara Vizzuso, Jacopo Cerasani, Fabio Mosca, Gian Vincenzo Zuccotti

**Affiliations:** 1 Department of Health Sciences, University of Milan, 20154 Milan, Italy; elvira.verduci@unimi.it; 2 Department of Pediatrics, Vittore Buzzi Children’s Hospital University of Milan, 20154 Milan, Italy; sara.vizzuso@unimi.it (S.V.); gianvincenzo.zuccotti@unimi.it (G.V.Z.); 3 Department of Clinical Sciences and Community Health, University of Milan, Via San Barnaba 8, 20122 Milan, Italy; giulia.vizzari@unimi.it (G.V.); jacopo.cerasani@unimi.it (J.C.); fabio.mosca@unimi.it (F.M.); 4 Fondazione IRCCS Ca’ Granda Ospedale Maggiore Policlinico NICU, Via Commenda 12, 20122 Milan, Italy

**Keywords:** breastfeeding, nutrition, obesity, microbiome, health outcomes

## Abstract

The benefits of human milk for both mother and infant are widely acknowledged. Human milk could represent a link between maternal and offspring health. The triad mother-breast milk-infant is an interconnected system in which maternal diet and lifestyle might have effects on infant’s health outcome. This link could be in part explained by epigenetics, even if the underlining mechanisms have not been fully clarified yet. The aim of this paper is to update the association between maternal diet and human milk, pointing out how maternal diet and lifestyle could be associated with breast-milk composition, hence with offspring’s health outcome.

## 1. Introduction

In accordance with the World Health Organization (WHO), the American Academy of Pediatrics (AAP), and the European Society for Pediatric Gastroenterology, Hepatology and Nutrition (ESPGHAN), human milk represents the normative feeding for infants during the first six months of life and later, in addition to the complementary feeding [1,2]. In fact, human milk meets the infants’ specific nutritional needs and leads to an adequate growth and functional development [3,4,5,6]. Increasing evidence has shown that breast milk provides not only nourishment for infants but also it reduces the risk of developing several diseases through the provision of bioactive factors including the occurrence of noncommunicable diseases such as obesity, type 2 diabetes mellitus, and cardiovascular disease later in life [7]. Breast milk allows mother-infant signaling within a closely linked system comprising the “mother-breast milk-infant” triad. Remarkably, variations occurring in each component of the triad appear to modulate the trajectory of infant development and maternal health [8].

We conducted a narrative review in order to provide an update on the available evidence regarding the association between maternal diet, lifestyle, and human milk composition. We searched the PubMed database for articles relating to breast milk using specific keywords such as breastmilk, human milk, maternal diet, nutritional, offspring outcome, microbiome. With regard to the maternal lifestyle we have chosen to focus on the most discussed topics in the recent literature, such as smoking, obesity, and plant-based diet. Preference was given to the sources published within the past 5 years. We included randomized controlled trials, cohort studies, systematic reviews, and meta-analysis. On the other hand, preclinical studies were excluded.

## 2. Nutrition during Pregnancy and Lactation

### 2.1. Energy Requirement

The energy requirement of a healthy, normal weight woman might be moderately increased during pregnancy, depending on pregnancy stage. At the same time, all women should maintain an active and healthy lifestyle, avoiding smoking and preventing excessive weight gain [9]. In fact, excessive as well as deficiency intake of macronutrients could be very harmful for mothers, fetal development, and for both later health outcomes. On the other hand, only a small increase of energy intake is necessary for milk production during lactation [10,11]. It is well known that higher demands of energy during pregnancy need a moderate increase in the energy value of the diet. However, over alimentation and overweight are associated with an increased risk of spontaneous abortion, gestational diabetes, pre-eclampsia, and also with infants’ development of type 2 diabetes and obesity during adult age [10,11].

In order to support fetal development and promote infant growth, daily energy value should be increased by about 70 kcal/day during the first trimester of pregnancy, 260 Kcal/day in the second trimester, and 500 Kcal/day in the third one and also during the first six months of exclusive breastfeeding, as recommended by the European Food Safety Authority (EFSA) [12]. Moreover, the energy cost of pregnancy is not equally distributed. In fact, energy deposition as protein occurs primarily in the third trimester (80%), and energy deposition as fat is based on rate of weight gain [13].

### 2.2. What about Macronutrients?

Protein intake could play an important role in fetal growth. As a matter of fact, low assumption of protein during pregnancy could influence both the weight and length of the newborn, as well as a high intake of protein may be negatively related with fetal development [14]. As a result, international guidelines agree in suggesting that the maternal protein daily intake increases by 26 g/day in the third trimester of pregnancy and by 21 g/day in the first semester of exclusive breastfeeding and by 14 g/day later, if breast milk still represents the main part of infant’s diet [9].

In our knowledge, a high protein intake during infancy is associated with an increased secretion of insulin and insulin growth factor (IGF-1) and so, with a greater early weight gain and with later obesity [15]. Even if breastfeeding has been related with a lower risk of future obesity, Forsum et al. showed that high maternal intake of proteins may be associated with higher levels of protein in breast milk [16]. In addition, Grunewald et al. in an observational study suggested that interindividual variations in human milk protein contents may contribute to modulate infant growth and lead to excessive weight gain even during full breastfeeding [17,18].

One of the main sources of energy is represented by lipids. During pregnancy and breastfeeding, an optimal lipid intake is possibly important for infant’s neurological and retinal development [19]. It is currently known that the quality of fats, rather than their total amount, plays a fundamental role. Most studies indicate a positive correlation between the fatty acid (FA) compositions in maternal diet and in breast milk [20], even if few other researchers did not show any association [21]. Moreover, there is high evidence of the role played by omega-3-polyunsaturated fats during pregnancy—in particular, docosahexaenoic acid (DHA) and arachidonic (ARA) acid [19]. According to recent literature, only fatty acids and vitamins profiles of human milk appear to be influenced by maternal diet [22,23].

Some authors studied the supplementation of DHA related to the psychomotor neurodevelopment in early life [24,25] and its possible association with lower risk of prematurity and post-partum depression [22,26,27,28,29,30]. Low blood levels of DHA were associated with low consumption of fish rich in omega-3, for example in vegetarian women [31]. In fact, in most studies, the use of fish oil supplementation during pregnancy and lactation was associated with higher levels of DHA [31,32,33]. The use of newly analytical methods, combined with appropriate bioinformatics and lipidomic analyses offers major opportunities to explore the physiological roles of complex lipids in early life and to achieve further improvements in nutritional strategies [19,34].

Marita de Waard et al. carried on a systematic review with the aim of exploring the correlation between maternal diet during lactation and infants’ long-term health outcome [35] and discovered that ten different studies focused on the relationship between maternal supplementation of long-chain polyunsaturated fatty acid (LC-PUFA) and infant growth or later body composition, without evidence of secure and consistent short or long-term positive associations [36,37,38,39,40,41,42,43,44]. FA composition of breast milk varies based on different factors such as lactation stage, gestational age, maternal nutritional status, and maternal lipid storage, which is the main source of omega-6 [21]. In addition, recent studies have shown a correlation between maternal perinatal psychopathologies, such as depression, anxiety, stress, and human milk composition in terms of macronutrients and immune components, immunoglobulin A, hormones, and cortisol. Some recent studies has shown that lower levels of DHA in breast milk were found in those countries where the prevalence of maternal depression was higher, impairing the adequate neurodevelopment of the offspring [45].

Moreover, Hahn-Holbrook et al. in their observational study highlighted a link between higher level of omega-3 PUFAs in human milk and lower level of negative affectivity at 3 months of age for those infants who were fully breast-fed. The pathways through which omega-3 PUFAs influenced infant temperament are not known yet, but their anti-inflammatory properties or their faculty to regulate neurotransmission could be involved [46]. As a result of a possible close correlation between maternal diet and DHA and EPA composition of human milk, Hahn-Holbrook et al. have showed how the maternal diet can influence the levels of omega-3 PUFAs in milk and thus infant behavior, reducing the stress and sadness of the offspring [46].

Lactose reflects carbohydrate energy content and, after protein, it is the most represented component of human milk. It is associated with infant growth, and its total amount in breast milk seems not to be affected by maternal diet [47,48]. On the other hand, the amount and quality composition of human milk oligosaccharides (HMOs) vary substantially between women in accordance to different factors such as enzymatic activity, which determine the synthesis of fucosylated HMOs and other genetic and environmental factors, which are not completely understood nowadays [48,49,50,51,52]. A better understanding of the maternal factors that influence this variability could be important in terms of health prevention. Indeed, HMOs could influence gut microbiota development, infant health, cognitive development, and disease risk [49,53,54]. The potential role played by HMOs in modulating newborn’s metabolism and infant growth is currently under investigation. Bardanzellu et al., in their observational study, found that a decreased breast milk content of Lacto-N-fucopentaose I and 2-Fucosyllactose (2′-FL) appears to be protective against an excessive weight gain, while an increased breast milk content of Lacto-N-fucopentaose II could predispose to it [55]. In addition, Larsson et al. in an exploratory study found significant differences in HMO breast milk composition between a group of exclusively breast-fed infants with high weight gain compared to a group of infants with normal weight gain. Further studies are needed to identify the mechanisms underlining this correlation; however, the most plausible hypothesis is represented by the link between HMO and the infant microbiota. Gaining further knowledge into the association between the HMO breast milk composition and their functional implication is possibly important in view of their supplementation in infant formulas to optimize the intestinal microbiota and positively influence infant growth [56].

Research has focused on the differences between the preterm and term human milk. Most recent studies report that macronutrient composition changes are more related to postnatal time than to gestational age [57]. Preterm milk is characterized by a higher total amount of protein than in term one; however, an overall decrease in total protein content during lactation is observed [57]. On the other hand, fat and energy density appears to remain constant over lactation [58]. With regard to the lactose content, it is higher in preterm than in term milk but maintains stable concentrations over time [59]. Interestingly, Fischer Fumeaux et al., in a prospective cohort study, identified some gender differences, as human milk dedicated to male infants seemed to be richer in fat and energy [57].

### 2.3. Micronutrients

During pregnancy, micronutrients are possibly important not only for their biological activity but also for leading to their storage for both maternal and fetal needs [60]. For this reason, they could be supplemented [61].

#### 2.3.1. Iron and Other Minerals

In pregnant women, iron requirement increases progressively in accordance to fetal needs and in accordance with its storage in fetal tissues. For this reason, women are exposed to a greater risk of iron deficiency, which is associated with increased risk of fetal growth failure, preterm labor, low birth weight, and post-partum hemorrhages [62,63]. Furthermore, recent evidences found a relationship between maternal insufficient iron intakes and higher possibility of cardiovascular disease for the infants in adult age [64]. On the contrary, an excess of iron supplementation might be associated with maternal development of oxidative stress, lipid peroxidation, impaired glucose metabolism, and gestational hypertension [65].

Despite these evidences, as shown in the study of Mahdavi et al. [66,67,68], the level of iron in breast milk does not appear to be related to the dietary intake, even if iron supplementation during lactation resulted in improved transferrin receptor levels and hematocrit [69].

Regarding other minerals, an adequate intake of calcium during pregnancy could play a role in fetal growth and development, even if its breast milk level is not substantially influenced by maternal diet intake [70].

In addition, iodine low intake during pregnancy may be associated with both maternal and infant negative outcomes, particularly regarding infant neurodevelopment [71]. 

#### 2.3.2. Vitamins

Since there is a high prevalence of insufficient maternal levels of vitamin D, doctors recommend its supplementation during pregnancy and breastfeeding [72]. De Regil et al. in a Chocrane review [73] demonstrated that an adequate intake of vitamin D was associated with a minor risk of pre-eclampsia, prematurity, and low birth weight. On the contrary, insufficient levels were associated with low birth weight, altered bone development, respiratory infections, and allergic diseases in childhood [9,73]. Czech-Kowalska et al. [72] carried out a prospective randomized controlled trial and focused their attention on the supplementation of vitamin D during breastfeeding both in mother and in infants. Body composition and bone mass assessed with dual-energy X-ray absorptiometry (DXA) three weeks, three months, and six months after delivery were not significantly different between infants of mothers receiving 1200 IU/d of cholecalciferol and those of mothers receiving 400 IU/d [72]. On the other hand, other studies showed that a higher dose of Vitamin D3 supplementation (6400 IU/day) was safe for both mother and child and resulted in a higher maternal level of 25-hydroxy-D [74]. Moreover, Basile et al. in a randomized controlled trial, demonstrated that a higher dose of vitamin D supplementation (4000 IU/d) was more effective in increasing the breast milk level of vitamin D compared with a lower dose (2000 IU/d) [75]. Furthermore, the relationship between inadequate levels of vitamin D and cardiovascular disease, hypertension, and diabetes has been described [76].

As for folic acid, there is strong evidence to support maternal supplementation in order to prevent neural tube defects [73,77].

Moreover, a positive correlation between maternal consumption of vitamin C and A and adequate levels in breast-milk was found in some observational studies, as shown by Kodentsova et al. [78], Salmenpera [79], Lietz et al. [80], and Da Silva et al. [81]. In fact, vitamin C is a milk antioxidant [82,83], and vitamin A plays a possibly important role in vision, intercellular communication, cell growth, and cell differentiation [81].

#### 2.3.3. Phytochemicals

Maternal diet can influence the offspring outcome, especially in terms of development, through the action of secondary plant metabolites commonly named as phytochemicals, such as flavonoids and carotenoids. Phytochemical intake occurs through the consumption of fruits and vegetables. Many of them could be antioxidant and anti-inflammatory agents and could reduce the risk of some chronic conditions (heart diseases, cancer, diabetes, and neurodegenerative disorders) [84]. Thus, adequate supplementation of maternal diet during pregnancy and lactation with fruit and vegetable is possibly important to guarantee an optimal concentration of flavoinoids and carotenoids both in maternal serum and breast milk [85]. Vishwanathan et al. in an observational study demonstrated that lutein is the predominant carotenoid in the brain, and its deposition in the human retina occurs early in life [85]. Zielinska et al. in their observational study showed a positive correlation between the concentration of omega-3 LC-PUFA and carotenoids in breast milk and infant motor and brain structural development [86]. Flavonoid contribution to infant’s oxidative stability is less clear [86].

## 3. Human Milk: A Contribution to the Development of Infant Gut Microbiota and Immunity

**Figure 1:**
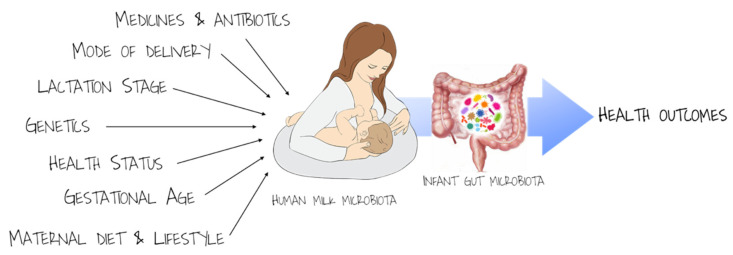
Modulation of infant gut microbiota via mother gut and human milk microbiota.

Several hypotheses have been formulated in order to explain the complexity and great diversity of bacteria contained in breast milk [87]. The dynamic cycling of bacteria from maternal commensal skin flora to infant mouth flora is one of the identified mechanisms [88]; however, this retrograde flow does not fully explain the diversity of human milk microbes. Therefore, an entero-mammary pathway has been hypothesized, whereby maternal intestinal bacteria migrate to the mammary glands via an endogenous cellular route during pregnancy and lactation [89]. Thus, the modulation of maternal gut microbiota during pregnancy and lactation could have a direct association on infant health. Several perinatal factors could influence microbial transfer from mother to infant via breast milk. All the factors that could modify maternal microbiota of skin, oral cavity, vagina, and gut may contribute to the modulation of human milk microbiota, including the lactation period, the mode of delivery and gestational age, the use of antibiotics or other medicines, the maternal dietary habits, and nutritional status [90] (Figure 1).

In particular, it is known that the gut microbiome composition differs in healthy and obese people; therefore, an aberrant microbiome can be vertically transmitted from an obese mother to her infant. Mother-to-newborn transmission of microbiota might be a causal factor underlying obesity’s transmission [91,92].

Not only the breast milk microbiota but also the oligosaccharides and other components of human milk, including Immunoglobulin A (Ig A), can contribute to the composition and diversity of the infant gut microbiome [93].

Human milk-associated microbes are among the first to colonize the infant gut and may help to shape both short- and long-term infant health outcome [94]. Accordingly, a recent meta-analysis showed that exclusive breastfeeding, especially longer than 2 months from birth, was associated with a more stable gut bacterial taxa composition and reduced diarrhea-associated microbial dysbiosis [95]. The critical window of immune development and the community types may induce metabolic alterations, leading to differing immune phenotypes and long-term health outcomes [46,88,96,97,98]. Crosstalk between host cells (e.g., intestinal brush border cells or immune cells) and the colonizing microbiota is likely to be critical for metabolic development and the programming of body immune system in infants [88,93].

## 4. Maternal Lifestyle and Nutritional Status during Pregnancy and Lactation and Later Health of Offspring: Some Traps

### 4.1. Tobacco Smoking

It is widely reported that maternal smoking during pregnancy and breastfeeding may be associated with negative outcomes of infants both at birth and later in life [99,100,101].

In particular, both the quantity and quality of breast milk might be negatively influenced by smoking just more than ten cigarettes per day [102]. Banderali et al. performed a descriptive review about associations of parental smoking during pregnancy and breastfeeding [103]. Recent observational studies showed a relationship between maternal smoking during pregnancy and the adverse outcomes at birth, such as low birth weight, prematurity [104,105], and negative outcomes on fetal brain development [100]. According to some authors, maternal tobacco could be associated with mammary glands synthesis and the secretion of DHA into breast milk [106,107]. Other observational studies demonstrated that fetal growth impairment might be also influenced by epigenetic factors through DNA methylation of particular genes, such as Cytochrome P450 1A1 (CYP1A1) promoter [108,109,110].

Recent evidences highlighted that maternal smoking during pregnancy was associated with increased risk of Sudden Unexpected Infant Death [111]. Moreover, it could be associated with increased risk of infants’ overweight, obesity, and metabolic diseases later in life [112,113,114,115]. Furthermore, some authors reported that there might be an association between hypertension and maternal smoking during pregnancy [116]. Last but not least, exposure to maternal tobacco was demonstrated to be related to higher incidence of respiratory tract diseases, such as airway hyper-responsiveness, wheezing, asthma, impaired lung function, and bronchitis [117]. Recent literature focused on the possible correlations of second- and third-hand smoking exposure on children outcomes [103,118].

Despite all these possible correlations, breast milk still remains the best feeding, even if the mother continues to smoke [119].

### 4.2. Obesity

Obesity represents one of the major public health issue all over the world. More than 20% of children in Europe and more than 30% in the United States suffer from obesity or overweight [35].

Obesity in childhood increases the risk of developing cardiovascular or metabolic diseases early in life. The fetal-infant programming hypothesis tried to explain how to prevent obesity in childhood by modifying the maternal diet and therefore the fetal exposure to excessive nutrients intake [120,121,122,123].

Over alimentation and overweight during pregnancy are associated with an increased risk of spontaneous abortion, gestational diabetes, and pre-eclampsia [10,11]. Moreover, children of overweight or obese mothers are at greater risk of having a high birth weight for gestational age, a rapid weight gain in the first year, and becoming obese in adulthood (Table 1).

Thus, special attention might be paid to lifestyle interventions, such as education and behavioral counseling related to diet and physical activity, that can be applied both during pregnancy and breastfeeding [124].

The correlation between breastfeeding and reduced risk of obesity later in life has been widely discussed in order to prevent rapid acceleration of growth during infancy and reducing the deposition of adipose tissue [125,126,127]. Haschke et al. [128] examined data from three randomized controlled trials, with the aim of exploring the association between maternal obesity and faster growth of their breast-fed infants [129,130]. Moreover, the authors focused on the effect of low-protein formula on infant growth. Results showed that infants fed with low protein amount formula may grow slower than those fed with high protein content formula, and this might represent a strategy to reduce the risk of obesity later in life [131,132,133].

In accordance, Inostroza et al. in a randomized double-blind study demonstrated that breast-fed infants of obese mothers may show a more rapid growth than infants of normal-weight mothers, particularly during the first six months of life [129].

Recent evidences confirmed that breast-milk composition was influenced by maternal nutritional status. Some studies analyzed the breast-milk composition of healthy and obese mothers and demonstrated the presence of different amounts of fatty acids, protein, and calories [134,135,136]. In particular, Leghi et al. in a recent systematic review highlighted an association between maternal obesity or overweight and fat and lactose concentration in human milk depending on different lactation stage; on the other hand, no correlation was found with protein concentration [92]. De Luca et al. aimed to compare breast-milk composition of obese versus normal-weight mothers. In their cross-sectional observational study, they found a higher amount of leptin in breast milk of obese mothers, whereas breast milk was not different in terms of protein, lipid, and carbohydrate composition and volume [137,138]. Leptin content has also been positively correlated with higher weight gain in infants and increased adiposity as far as 12 months of lactation. In fact, researchers have found a correlation between breast-milk leptin and infant serum leptin, and between infant serum leptin and both infant BMI and weight [139]. Kirchberg et al. identified different metabolic clusters in a cohort of breast-fed infants of 6 months old. They emphasized the heterogeneity of metabolic patterns characterizing breast-fed infants; however, further studies are needed to examine the potential role of these data to predict the risk of obesity in childhood [140]. In addition, a recent cohort study by Samuel et al. suggested that the HMO breast milk composition varies depending on different factors such as pre-pregnancy body mass index (BMI), mode of delivery, and parity. In particular, pre-pregnancy BMI might influence maternal HMO glycosylation and could contribute to the increased obesity risk in children of obese mothers [141].

### 4.3. Plant-Based Diet

There is no consensus regarding the relationship between vegetarianism and health outcomes of both mothers and babies. The American Academy of Nutrition and Dietetics [142,143] argued that a well-balanced plant based diet is safe and supports sustainable growth and development in all age groups. On the contrary, the Swiss Federal Commission for Nutrition [144], the German Nutrition Society (DGE) [145], and ESPGHAN [146] do not recommend the adoption of a vegan diet during pregnancy or lactation, in order to avoid the development of nutritional deficiencies. Moreover, the importance of adequate supplementation and nutrition counseling for these groups of women has been highlighted [146,147].

In addition, plant-based diets during lactation still raises doubts about human milk donations. According to the European Milk Bank Association (EMBA) Guidelines, mothers following a vegan diet without an adequate supplementation should not donate their milk [148].

Vegetarian and vegan diets are associated with major risk of nutritional deficiencies compared to omnivorous one, but update evidences highlight that if adequately supplemented, vegetarian and vegan diets could be considered safe for mothers and the offspring health during pregnancy and lactation [9,149,150]. Plant-based diets have been reported to contain more folate, fiber, antioxidants, and carotenoids and less saturated fatty acids, protein, and cholesterol [151], on the other hand, a low content of essential micronutrients especially in terms of iron, zinc, vitamin B12 [152], vitamin D, omega-3 (n-3) fatty acids, calcium, and iodine has been described inn vegetarian diet [153]. For this reason, micronutrient deficiencies might not be underestimated [149].

### 4.4. Chemical Residues

Unfortunately, breast milk is not pristine, and this is due to environmental pollution. Contamination of human milk is widespread [154]. Polychlorinated biphenyls (PCBs), dichlorodiphenyltrichloroethane (DDT) and its metabolites, dioxins, dibenzofurans, polybrominated diphenyl ethers (PBDEs), and heavy metals are the main chemical contaminants most commonly found in breast milk. Exposition to lipophilic toxic chemicals occurs in everyday life through the air, water, and food, both at home or at the workplace [155]. Generally, pollutants enter breast milk by passive transfer from maternal plasma, and their concentration is proportional to their lipophilicity and solubility [154]. Despite its importance, few studies have been conducted on this topic, but the data obtained so far would seem encouraging. Van den Berg et al. conducted a global survey indicating that the human exposure to polychlorinated biphenyls and dioxin-like compounds is still above those considered toxicologically safe for the fetus and breast-fed infant [156].

## 5. Conclusions

Breast milk represents a complex and dynamic system that allows mother-infant communication and signaling. The components of the “mother-breast milk-infant” triad are closely connected to each other, and every single variation could affect the trajectory of infant development or maternal health [8]. Taking care of women’s health, in terms of diet and lifestyle, during the preconception period, pregnancy, and breastfeeding could represent a prevention strategy in terms of improving the offspring health [157]. Nowadays, the increased percentage of women who follow elimination diets by choice or by necessity underlines the importance of providing specialist care in order to prevent malnutrition and the adverse associations with maternal health and infant’s growth and development [151].

As this is a narrative review, this study does not provide an exhaustive account of all available literature, but it attempts to give a broad overview of existing evidence published on the triad mother-breast milk-infant. Moreover, a limitation of this review is that, despite a Mendelian randomization study and ten clinical trials, the vast majority of the considered studies were observational ones so, by their nature, they run the risk of containing confounding biases. The associations noted in observational studies such as between breastfeeding and outcomes might be actually due to the influence of the social determinants of health. The social determinants of health include opportunities for education and employment, level of income, ethnicity, race, access to housing and affordable utilities, access to health care, social and community support, early childhood education, neighborhood crime rates, and access to transportation and leisure activities [158]. Improving public health passes from health equity and measures to reduce disparities should be integrated into health programs and services [1,2] [159,160]. Health and social workers need to understand the importance of social determinants of health and to work together to make available the best health opportunities for all the population. Addressing the social determinants of health could represent a winning strategy for promoting more equitable health outcomes for patients, families, and communities [159]. On the other hand, even though some social determinants of health can be modifiable by supportive environments or clinical preventive practices and health programs, many of these when considered at the maternal level can result from upstream and insidious structural forces at play that go beyond maternal choices and immediate social and economic opportunities [159]. Social determinants of health, even if not modifiable and not dependent on maternal choices, could play a role on mother’s health and infant development individually and on the mother-breast milk-infant triad. In a recent cross-sectional study conducted by Gea Horta et al., factors associated with nutritional outcomes in 3676 mother-child dyads at the household level were analyzed, and it was pointed out that lower maternal education levels and living in inadequate households were associated with the double burden of malnutrition and the lack of breastfeeding was associated with maternal overweight [160]. Moreover adverse social determinants could affect breastfeeding initiation and early cessation as evaluated in a cohort study by Newhook at al. [161]. In fact, a socioeconomically disadvantaged population with low levels of education and income seems to be much less likely to breast-fed than their peers with higher levels of income and education [161]. In this kind of population, community support systems, such as trained health workers, lactation consultants, and community leaders, become essential to sustain breastfeeding [162].

By the end, social determinants of health appear to modulate maternal physiology and nutritional status and thus breast milk composition, which, in turn, could be associated with infant growth and health outcomes. Further studies are needed to achieve better knowledge about the mechanisms underlining this association [163] and define the prevention and therapeutic strategies aimed to promote infants’ growth, development, and health.

## Figures and Tables

**Table 1:** Effect of maternal obesity on the mother-baby dyad.

Maternal Obesity	
Side Effects on Mother	Side Effects on Infant
spontaneous abortion	Type 2 diabetes
gestational diabetes	Obesity
pre-eclampsia	Cardiovascular diseases

## Data Availability

Not applicable.
